# In an Emergency Setting, What Is the Best Intramuscular Pharmacological Treatment to Give to an Agitated Geriatric Patient?

**DOI:** 10.7759/cureus.25382

**Published:** 2022-05-27

**Authors:** Kevin Malone, Sall Saveen, Janice Hollier

**Affiliations:** 1 Biomedical Engineering, Louisiana State University Health Sciences Center, Shreveport, USA; 2 Psychiatry, Louisiana State University Health Sciences Center, Shreveport, USA

**Keywords:** riker sedation-agitation scale, geriatric psychosis, geriatric psychiatry, geriatric, emergency psychiatry, emergency medicine physician, emergency

## Abstract

Herein, we describe an agitated geriatric patient who suffered an adverse outcome due to treatment for agitation in an emergency setting. This led to the prompt review of the current literature on best-practice medication to use in this population. After careful review, the authors recommend olanzapine as the first-line medication for agitation due to its lower risk-averse effect profile when compared to other medications used for this patient population.

## Introduction

Agitated geriatric patients frequently present to the emergency department and acute psychiatric units in need of treatment. In older adults, agitation is a complex syndrome that is associated with multiple psychiatric and medical conditions and comorbidities. In the United States, by 2060, the number of individuals over the age of 65 is expected to increase to almost 98 million older adults [1]. Additionally, patients with dementia are expected to double within the next 30 years; these patients are frequently agitated, up to 50% display hallucinations or paranoia, and have a higher rate of emergency room utilization [1]. While this is a common presentation in the geriatric population, there are few guidelines as to the medical and pharmacological treatment of this patient population. The currently available medications used for treating agitation in geriatric patients have questionable efficacy data supporting their use and notable side effects. There is a need to provide a clear explanation of commonly used treatments, their risks, and overall recommendations. Herein, we describe an agitated geriatric patient who suffered an adverse outcome due to treatment for agitation in an emergency setting. These medications are still commonly prescribed as first-line treatments in elderly agitated patients. This led to the prompt to review the current literature on best practice medication to use in this population.

## Case presentation

A 78-year-old caucasian male with a past psychiatric history of major neurocognitive disorder presented to the emergency department for emergent evaluation under protective custody from a nursing home. The staff at the nursing home stated that the patient threatened to hang himself, destroyed property, threatened staff, soiled himself, and refused to change his underwear. The nursing home staff stated that they first tried to redirect the patient. Unsuccessful attempts required the patient to receive haloperidol 5 mg intramuscular (IM) once for severe agitation and psychosis at the nursing home. The patient continued to be aggressive after several doses and was transferred to the emergency department. The patient was placed on the physician's Emergency Certificate status in the emergency department. The patient did not have a history of drug abuse or physical illness. His physical health was optimal with no major comorbidities. It was not until the age of 70 that the patient was diagnosed with a major neurocognitive disorder. The patient’s mother was diagnosed with Alzheimer’s disease at the age of 68. No other family genetics were of significance in regards to a cognitive disorder.

The patient exhibited the presence of suicidal and homicidal ideation but denied auditory and visual hallucinations. He was oriented to person, and he knew the year, but could not recall the name of the hospital although he knew it was a hospital. He was uncooperative and agitated but maintained good eye contact. Pacing his room in the emergency department, he stated, “I don’t need to be here.” His speech was delayed and his mood was "irritable" with blunted affect. His thought process was vague. No ruminations, rituals, or phobias were appreciated. Likewise, no persecutory, paranoid, or grandiose delusions were appreciated. He had a normal gait with normal swing and stance (no stiff gait, extrapyramidal symptoms (EPS), tics, tremors, psychomotor agitation, or psychomotor retardation appreciated). Musculoskeletal showed full range of motion and the patient was ambulating independently with no difficulties and without needing assistance. Labs and EKGs were insignificant. CT scan of the head showed mild cortical and moderate ischemic changes noted in the gray-white matter, with no acute changes.

Intervention

In the emergency department, the patient continued to show signs of agitation and aggression. The patient had suicidal ideations with a plan of hanging himself. Further, he had homicidal ideations and was threatening staff. Psychomotor agitation was present, with the patient being aggressive, agitated, and destroying property. He denied auditory and visual hallucinations. Haloperidol 10 mg IM every four hours as needed for severe agitation and/or psychosis was ordered, and he was given two PRN doses. Further, the patient continued to be severely agitated and one dose of ziprasidone 20 mg IM was ordered and given prior to his transfer to the geriatric inpatient psychiatric facility. The patient was deemed stable and was transferred to a psychiatric facility. The total medication the patient received prior to the transfer was one dose of haloperidol 5 mg IM in the nursing home, two doses of haloperidol 10 mg IM in the emergency department, and one dose of ziprasidone 20 mg IM in the emergency department.

Outcome

Once the patient arrived at the geriatric facility, on physical exam, he was noted to be drooling with his tongue protruding from his mouth. He was unable to retract his tongue. The patient was difficult to understand. Behavior was restless and uncooperative, and the patient was not able to maintain eye contact. His speech was incoherent and slowed with delayed responses, and his mood was "unstable" and irritable with flat affect. His thought process was illogical and disorganized. He appeared suspicious and paranoid but denied suicidal ideations, homicidal ideation, auditory hallucinations, and visual hallucinations. The patient was alert and oriented to name only but not year, month, or place. He incorrectly believed the month was October and the year 2024. Further, his memory was not intact to recent and remote events. The patient was unable to recall why he was first brought to the hospital and then to the geriatric facility. His attention and concentration were poor. Similarly, his insight and judgment were poor and limited. The patient presented with extrapyramidal symptoms, displaying a rigid stooped posture and a slow, shuffling, and stiff gait. His musculoskeletal movements had a diminished range of motion and a significant amount of cogwheeling. The patient took several weeks to return to baseline.

## Discussion

Within this paper, we described the case report of a poor outcome of a patient who was given a sedative in the emergency setting. The patient was initially prescribed haloperidol and ziprasidone, which caused poor outcomes for this patient. Both haloperidol and ziprasidone are still commonly prescribed as an initial approach medication for older adults in the emergency setting. While there are often better alternatives, preference is still often unduly given to medications familiar to staff and typically prescribed to general populations.

Except for patients who present with a primary psychiatric or painful condition, pharmacologic intervention for agitation should be restricted to those patients who demonstrate severe agitation and who are at risk of harming themselves or others [1-2]. Further, such pharmacological management should facilitate time‐sensitive diagnostic imaging, procedures, or other treatments [1-2]. The currently available medications used for treating agitation in geriatric patients have questionable efficacy data supporting their use and notable adverse effects. For patients for whom a pharmacologic intervention is necessary, oral therapies are preferred because they typically have fewer side effects and longer duration. Nevertheless, oral treatments have limitations, including requiring patient cooperation and a slower onset of action. A lower dose should be given in older adults than is generally used for younger adults due to changes in age-related metabolism and pharmacokinetics as well as the risk of polypharmacy [3]. The choice of which medication to administer to the elderly population is dependent on the particular patient. Additionally, there is no determined consensus on the ideal medication for elderly patients with agitation within the current literature [4]. It is the opinion of the authors of this article that olanzapine is an excellent first choice when acceptable.

Initial approach to the elderly patient with agitation

First, identify the underlying cause(s). Multiple factors are common, and priority should be given to more severe cases; however, all should be addressed by the provider. It is helpful to identify the most likely reason that the patient is in acute agitation.

As a starting differential for an agitated elderly patient, acute agitation can be expressed in several conditions, including medical, psychiatric, and substance-induced alterations (Table 1) [5]. A differential of possible causative factors may help with guiding treatment in the short and long term after the initial agitation has resolved. In most cases, medications used for agitation have different efficacy based on the presenting illnesses. Therefore, forming a differential of underlying illness is pertinent.

**Table 1:** Starting differential Source:  [6-7]

Differential for Altered Mental Status	
Neurological	Toxicological
• Stroke	• Anticholinergic agents
• CNS tumors	• Serotonergic agonists
• Intracranial hemorrhage	• Benzodiazepines
• Meningitis	• Steroids
• Encephalitis	• Neuroleptics
• Psychiatric	• Alcohol abuse
• Bipolar disorder	• Alcohol withdrawal
• Schizophrenia	• Cannabis use
• Delusions	Infectious
• Metabolic	• Systemic infections
• Dementia	• Fever-Related delirium
• Lewy Body	• Sepsis
• Parkinson’s	Deliriogenic Factors
• Trauma	• Physical needs not met
Electrolyte abnormalities	• Disorientation
• Hyper/hypoglycemia	• Environmental factors
• Hyper/hyponatremia	

Additionally, it is good practice to assess initially for major comorbid conditions, which may affect the acute administration of medications and lead to worse outcomes. The comorbidities include QTc prolongation (If known, questionable >475 and contraindicated >500); History of sedating medications and recent administration (Stacking sedation); Fluid volume status, orthostatic hypotension; Any metabolic disorders; History of current uncontrolled heart failure/cardiac disease.

Nonpharmacological management

Agitation should be managed with nonpharmacologic strategies first, whenever possible if there is no immediate risk of harm to the patient or staff. When effective, these interventions will have the advantage of decreasing adverse events in this frail population. A good practice is training staff appropriately in nonpharmacological management to prevent the initiation of agitation whenever possible.

Aggression, exit-seeking, and resistance to care should always be considered in elderly agitated patients with unmet needs. Ensuring that patients are comfortable and removing deliriogenic triggers common in the emergency setting may often be enough to resolve an agitated patient. Resolving the physiologic needs within the lower orders of Maslow’s hierarchy, such as the need for food, water, comfort, toileting, sleep, visual/auditory impairment requirements, and freedom from pain/nausea, may improve the patient’s demeanor and mood. Reorientation and control of light, i.e., keeping typical day-night cycles, may help reduce confusion. Removing triggers such as loud noises, alarms, background distractions, and other noxious stimuli familiar to the emergency setting may reduce agitation. Unnecessary tethers should be removed from the geriatric patient when possible, which may not only limit their mobility and frustrate and confuse them but can also decrease the risks of falls and injury. Additionally, the patient may be able to be verbally de-escalated by healthcare providers with a calm demeanor, reorientation, distraction, and reassurance. In this patient population, reorientation or calming strategies, such as the involvement of family members and volunteers or the use of sitters, may help reduce agitation in these patients and prevent injury. When possible, maintaining consistency and structure in setting and schedule decreases the need for the patient to adjust to new, potentially anxiety-inducing situations or environments [8-9]. Other nonpharmacological methods have varying degrees of supporting evidence, especially within the acute setting. Although physical restraints can act as an asset in the emergency setting to prevent harm to staff or the patient, they should be reserved in only extreme circumstances that are dangerous and refractory to treatment. Using restraints could cause harm to the elderly patient physically and increase any agitation; restraints are therefore not recommended for initial use in the elderly population [1,10].

The creation and maintenance of a “no-fail” environment in geriatric areas minimize the need for patients to adapt to their surroundings. Prevention of agitation, wandering, or escape-seeking behaviors includes the placement of a red “STOP” sign on the exit door, placing paintings of furniture in front of a door, and painting the door, which includes the doorknob, the same color as the surrounding walls [1,11]. Validation therapy involves verbalizing acceptance of a patient’s personal experiences. Several early studies suggest that validation therapy helps decrease depression and behavioral disturbances in older adults who present with dementia. A more recent case-control study suggests that validation therapy may reduce the frequency and severity of the psychological and behavioral symptoms of dementia [11].

Pharmacological management

Pharmacological treatment may be indicated when non-pharmacological strategies fail to manage agitation effectively. In assessing pharmacological intervention, benefit-to-risk ratios should be evaluated. Whenever possible, informed consent from the patient or legal surrogate should still be obtained and documented.

When prescribing medications, a drug's indications, contraindications, and onset time are important considerations. Although the time in which a drug takes effect varies significantly based on the type of medication used, the average onset time for most intravenous medications is 5 minutes, for most intramuscular medications, it is at least 15 minutes, and for most oral therapies, it is at least 30 minutes. Failure to recognize the adequate onset of action of therapeutic effect could lead to either mixing medications or dose stacking, both of which increase the risks of adverse effects, particularly for older adults. See Table 2 for pharmacological interventions recommended by the American Psychiatric Association (APA).

**Table 2:** Relevant statements by the APA on pharmacological interventions Source: [5]

American Psychiatric Association (APA) on Pharmacological Interventions
1. Non-emergency antipsychotic medication should only be used for the treatment of agitation or psychosis in patients with dementia when symptoms are severe, are dangerous, and/or cause significant distress to the patient.
2. Recommend reviewing the clinical response to nonpharmacological interventions prior to non-emergency use of an antipsychotic medication to treat agitation or psychosis in patients with dementia.
3. Before non-emergency treatment with an antipsychotic is initiated in patients with dementia, the potential risks and benefits from antipsychotic medication be assessed by the clinician and discussed with the patient (if clinically feasible) as well as with the patient’s surrogate decision-maker (if relevant) with input from family or others involved with the patient.
4. In the absence of delirium, if non-emergency antipsychotic medication treatment is indicated, haloperidol should not be used as a first-line agent.
5. In patients with dementia with agitation or psychosis, a long-acting injectable antipsychotic medication should not be utilized unless it is otherwise indicated for a co-occurring chronic psychotic disorder.

Antipsychotics are frequently used in older adults. First‐generation antipsychotics, such as haloperidol and droperidol, have a different mechanism of action and adverse effect profile than second‐generation antipsychotics, such as olanzapine, risperidone, ziprasidone, and quetiapine. First‐generation antipsychotics primarily act on dopamine D2/D3 receptors, which are highly expressed in the basal ganglia. In contrast, second‐generation antipsychotics primarily block serotonin receptors and, to a lesser extent, block dopamine receptors [12-14]. Care should be observed for patients who have congenital or baseline prolonged QT intervals, channelopathies, and patients who receive other QT‐prolonging medications. Although all antipsychotics risk QT prolongation, first‐generation antipsychotic medications pose the greatest risk. For example, droperidol has a black box warning that notes the risk of QT prolongation and torsades de pointes seen in higher doses [15-16]. In addition, many first‐generation and second‐generation antipsychotics contain a black box warning regarding the increased risk of mortality in geriatric patients with dementia as a result of cardiac and infectious causes [17-18]. These antipsychotics are also associated with an increased risk of death or cardiac arrest in hospitalized older adults [19]. For commonly administered antipsychotics, the estimated number needed to harm ranges from 26 to 50. As a result, these medications should only be used when necessary [20]. Haloperidol is not routinely recommended for agitation in dementia due to its low efficacy and high risk of adverse effects [3,21]. After the Food and Drug Administration (FDA) issued a black box warning, the use of droperidol rapidly decreased. Consequently, there is sparse research on droperidol, including its side effects and efficacy, in the management of agitation in older adults or patients with dementia [15-16]. First‐generation antipsychotics have high rates of extrapyramidal side effects, such as acute dystonia, akathisia, pseudoparkinsonism, and tardive dyskinesia, because they blockade dopamine receptors. These antipsychotics should also be avoided in patients who have Parkinson’s disease or Lewy body dementia [14]. In general, second-generation antipsychotics are preferred over first-generation antipsychotics. Historically, there was debate over the preferred medication within this class for use in elderly agitated patients. FDA black box warnings currently warn against the use of atypical antipsychotics in elderly patients, especially those with dementia. This may have decreased the use of second-generation antipsychotics, even in patients who exhibit obvious psychotic signs and symptoms in addition to agitation [22-24].

Olanzapine is a second-generation atypical antipsychotic medication with a safer side-effect profile than conventional antipsychotics. Olanzapine is primarily a 5-HT antagonist and presents lower potency at the D1, D2, and α1 receptors. Olanzapine acts as an antagonist on dopamine D2 receptors, where it blocks dopamine from the post-synaptic receptor. Olanzapine loosely binds to the receptor and dissociates from it easily, which allows for normal dopamine neurotransmission. Like the D2 receptor, Olanzapine binds to the serotonin 5HT2A receptors in the frontal cortex as an antagonist [25-26].

For patients over the age of 13, the FDA has approved olanzapine for schizophrenia. Olanzapine is also approved for both mixed or manic episodes of bipolar disorder. Olanzapine can be safely used for acute agitation and delirium [27-29]. Elderly patients may often present with acute psychotic symptoms that need antipsychotic treatment. Olanzapine is one of the atypical antipsychotics with efficacy for psychotic symptoms and agitation, and it has a safer side-effect profile than typical antipsychotics [30].

Olanzapine has several benefits over other conventional typical and atypical antipsychotics. Generally, in elderly patients, olanzapine is very well-tolerated [31]. Olanzapine has shown greater efficacy in the outpatient management of behavioral and psychological symptoms of dementia (BPSD) with less need for follow-up administrations. Duong et al. found that in the treatment of BPSD, 2.5 mg and 5 mg doses of olanzapine IM appeared to achieve efficacy in about 80% of documented cases [28]. Meehan et al. found less need for the follow-up administration of olanzapine compared with others [32]. In elderly patients with a higher risk of dose-stacking, this is particularly noteworthy. Additionally, olanzapine is available both intramuscularly and orally, which allows a bridge from IM use to oral use in patients who need extended treatment. Madhusoodanan et al. demonstrated the safe use of olanzapine (5-20 mg/day) in 11 elderly patients aged 60-85 years with schizophrenia or schizoaffective disorders [33-35]. There seemed to be nearly no cardiac effects as seen with other antipsychotic medications and first- and second-generation antipsychotics [31]. Fewer extrapyramidal symptoms have been observed with olanzapine as compared with typical antipsychotics and most atypical antipsychotics at therapeutic dose ranges [36]. Of the extrapyramidal symptoms observed in atypical antipsychotics, olanzapine has been seen to have less EPS than quetiapine but similar rates as ziprasidone [37-38]. In general, olanzapine has a low potential for toxicity when prescribed alone. Orthostatic hypotension can be seen with single-dose use while weight gain and metabolic dysfunction can be seen with chronic use. Previous studies reported a prevalence of sedation and orthostatic hypotension using olanzapine intramuscularly [39]. Older adults experience a decreased level of consciousness and increased risk of falls. Only a few case reports found the adverse effect of hypotension with olanzapine toxicity as a result of higher doses taken with other medications. One report exists of a patient who overdosed by taking 560 milligrams of olanzapine with 280 milligrams of amlodipine and 6.4 grams of propranolol, which resulted in excessive hypotension, circulatory failure, respiratory depression, and eventual coma [26]. It is therefore recommended to avoid prescribing olanzapine with other medications that may cause hypotension and cardiopulmonary depression. Intramuscular olanzapine should not be used in conjunction with benzodiazepines and should be administered at a minimum of two hours post-administration of benzodiazepines. The use of oral administration of olanzapine over intramuscular administration may decrease this risk of hypotension.

The most common adverse effects of olanzapine use are potential weight gain and reduced insulin sensitivity. Studies are limited regarding these effects in older adults, as most metabolic studies were conducted on younger populations [32,40]. Although more research is needed on the elderly population, clinicians generally should use caution in prescribing this medication in conjunction with patients with obesity or diabetes mellitus. However, olanzapine is not an absolute contraindication for patients who are obese or have diabetes [40]. Monitoring blood glucose levels post-administration can mitigate potential risks [28-30]. Current research supports the use of olanzapine in treating elderly patients with agitation. It is then recommended to use olanzapine over haloperidol and ziprasidone for use in elderly patients. Both the intramuscular and oral administration of olanzapine seem efficacious in this patient population and offer safety advantages over other medications [36,41-42]. While the adverse effect profile of olanzapine seems to be greater than other medications of its class, it is still recommended to monitor vitals, level of consciousness, insulin sensitivity, and gait stability to prevent adverse events. In outpatient studies, quetiapine has demonstrable effectiveness on the behavioral and psychiatric symptoms of dementia. However, quetiapine is associated with a high rate of sedation [23,43]. Because quetiapine has been shown to have the least extrapyramidal side effects of the second‐generation antipsychotics, it is the preferred oral medication for patients with Parkinson’s disease or Lewy body dementia [25,44].

Ziprasidone may be an option in some cases; in particular, some clinicians prioritize ziprasidone for patients with severe pre-existing metabolic issues. These patients should be informed that Ziprasidone has a high rate of early treatment discontinuation, primarily due to a lack of efficacy or adverse effects, and that such risks may be lower with other antipsychotics [31,39]. Ziprasidone also has a more extensive adverse effect profile than Olanzapine, including known cardiac effects (increased QTc). However, Ziprasidone may be a suitable option in cases where initial treatment with olanzapine failed or brought about rare adverse effects and may be used as a follow-up medication.

For patients who have benzodiazepine or alcohol withdrawal or dependence, benzodiazepines are the first-line treatment [27]. In patients with Parkinson’s disease and Lewy body dementia, who require parenteral treatment of severe agitation, benzodiazepines may be preferable to antipsychotics because they do not cause dopamine receptor blockade and associated extrapyramidal side effects. When benzodiazepines are determined to be the most appropriate treatment, short‐acting agents (e.g., lorazepam and midazolam) should be preferred over long‐acting agents (e.g., diazepam). Benzodiazepines generally should otherwise be avoided as they are more likely to cause respiratory depression, prolonged sedation, falls, and delirium [36,45].

There is minimal research evaluating ketamine's adverse drug effects and efficacy in elderly patients even though ketamine is increasingly used for younger patients in the ED for acute pain and management of excited delirium syndrome. However, studies of ketamine at sub‐dissociative doses for pain in elderly patients indicate a high rate of side effects, including agitation and depersonalization. As a result, ketamine is not currently recommended for managing agitation in elderly patients [46-47].

For treating severe agitation in younger patients, administering combinations of agents (e.g., a “B52” combination of benzodiazepines, antipsychotics, and anticholinergics) is a common practice [48]. However, due to an increased risk of adverse drug effects, such combinations should be avoided for elderly patients [48]. In addition, intravenous benzodiazepines and intramuscular olanzapine should not be administered within two hours of one another due to the increased risk of cardiopulmonary depression and hypotension [49-50]. Adjunctive medications (e.g., benztropine, diphenhydramine, or promethazine) are sometimes prescribed to prevent extrapyramidal side effects, but these medications should generally not be administered to geriatric patients, who face an increased risk of developing adverse drug effects from the anticholinergic effects, including acute delirium. Table 3 lists the commonly used medications in agitated geriatric patients.

**Table 3:** Commonly used medications in agitated geriatric patients Source: [1] ADE: adverse drug effect

Medication	Typical Dose/Route	Minutes to Onset	Considerations and Precautions in Older Adults
Droperidol	5 mg IM	5–10	● Rapid onset ● Limited data for use in geriatric patients ● ADEs: QT prolongation, increased risk extrapyramidal side effects ● Avoid in Parkinson's disease and Lewy body dementia
Haloperidol	1–2 mg PO 1–2.5 mg IM	90–120 20–60	● Rapid onset ● Avoid higher IM doses (5-10mg) in this population, may cause prolonged side effects and somnolence ● ADEs: QT prolongation, increased risk extrapyramidal side effects ● Consider obtaining EKG when available ● Avoid in Parkinson's disease and Lewy body dementia
Risperidone	0.25–1 mg PO	30–120	● Greater efficacy in the outpatient management of BPSD ● ADEs: orthostatic hypotension, increased risk of falls especially in volume-depleted or frail patients.
Ziprasidone	10–20 mg IM	15–30	● ADEs: QT prolongation, orthostatic hypotension, increased risk of falls ● Strong caution against patients with uncontrolled heart failure, cardiac disease, intoxication, or volume-depleted/orthostatic patients.
Olanzapine	2.5–5 mg PO/SL 2.5–5 mg IM/IV	15–120 15–30	● Available as an oral disintegrating tablet ● Greater efficacy in the outpatient management of BPSD ● ADEs: orthostatic hypotension (IM>PO), increased risk of falls ● Avoid IM use within 2 h of IV benzodiazepines due to risk of hypotension and cardiopulmonary depression
Quetiapine	12.5–25 mg PO	30–120	● Greater efficacy in the outpatient management of BPSD ● Preferred oral agent in Parkinson's disease and Lewy body dementia ● ADEs: high‐risk of orthostatic hypotension, increased risk of falls, somnolence
Lorazepam	0.5–1 mg PO 0.5–1 mg IM 0.5–1 mg IV	10-15 5-10 5-10	● Preferred in alcohol or benzodiazepine withdrawal ● May be the preferred parenteral agent in Parkinson's disease and Lewy body dementia due to the lack of extrapyramidal side effects ● Rapid onset ● ADEs: paradoxical excitation may precipitate or worsen delirium ● Avoid IV use within 2h of IM Olanzapine due to risk of hypotension and cardiopulmonary depression ● May cause paradoxical excitement in this population ● Lorazepam is the preferred Benzodiazepine in this patient population.
Midazolam	2.5–5 mg IM	10-15	● Rapid onset ● ADEs: paradoxical excitation, may precipitate or worsen delirium ● Avoid IV use within 2 h of IM Olanzapine due to risk of hypotension and cardiopulmonary depression

Post-intervention care management

This is the most neglected portion and one of the most critical when administering medications in this class. Within the older population, regular post-sedation monitoring is of great concern given the vulnerability of elderly patients to adverse effects. APA guidelines call for monitoring of these patients [5]. Several factors may contribute to this, including a lack of specific guidelines on the frequency of observations required post-sedation, a fear from nursing and medical staff that regular examination of the patient may further agitate the patient, and perhaps a lack of awareness of the side effects of sedation. Studies have indicated a high rate of adverse events following pharmacological sedation, and thus there is always a need to closely monitor these patients [4].

Recommendations for care

Our recommendations include 1. Prevention of agitation and staff training can help reduce pharmacological interventions and in turn, adverse outcomes; 2. When agitation is present, identify the underlying causes of agitation and possible diagnosis. While multiple factors are common, one should prioritize but address them all; 3. Identify any complicating comorbid factors that may affect care; 4. Agitation should be managed with non-pharmacologic strategies first. Treat any underlying causes. Ensure physical needs are met, optimize patient comfort, and decrease the “deliriogenic” factors within the environment. Physical restraints should be avoided; 5. If non-pharmacologic strategies fail, treat using pharmacological strategies. Use oral agents if possible, “start-low, go-slow”; 6. Antipsychotic agents should be reserved for unremitting symptoms that threaten patient safety. Olanzapine is recommended over haloperidol and ziprasidone for use on elderly patients when indicated; 7. Always reassess the patient and monitor the patient looking for signs of adverse effects and over sedation. Remember this is a vulnerable population.

## Conclusions

In older adults, agitation is a complex syndrome, which is associated with multiple psychiatric and medical conditions and comorbidities. Although the impact of agitation on elderly patients, caregivers, and health care costs is significant, there is much that remains unclear about the causes, prevention, and treatment of agitation. Clinical interventions that use individualized and multidisciplinary best-practice approaches should be routine. Further research is needed to more rigorously test existing treatments and to develop new interventions for elderly patients with agitation. When possible, it is essential to prevent the use of pharmacological interventions as there is a higher risk of adverse effects in this population. When choosing a medication, it is also important to note the possible causative factor and any comorbidity that may affect the choice of medication. Olanzapine is recommended over other first- and second-generation antipsychotics as an initial medication in elderly agitated adults. It is imperative that proper monitoring of this population post-medication administration is done in all cases. The key to treating agitation with the least adverse events in this population involves good hospital policies and procedures in preventing agitation and using less invasive interventions.
